# The failure of success: four lessons learned in five years of research on research integrity and research assessments

**DOI:** 10.1186/s13104-022-06191-0

**Published:** 2022-09-24

**Authors:** Noémie Aubert Bonn, Raymond G. De Vries, Wim Pinxten

**Affiliations:** 1 Department of Healthcare and Ethics, Faculty of Medicine and Life Science, Hasselt University, Hasselt, Belgium; 2 Center for Bioethics and Social Sciences in Medicine, University of Michigan Medical School, Ann Arbor, USA

**Keywords:** Research assessment, Research integrity, Research culture, Publication pressure, Diversity, Precarity, System change

## Abstract

In the past 5 years, we captured the perspectives from a broad array of research stakeholders to better understand the impact that current approaches to success and research assessment may have on the integrity and the quality of research. Here, we translate our findings in four actions that are urgently needed to foster better research. First, we need to address core research structures to overcome systemic problems of the research enterprise; second, we must realign research assessments to value elements that advance and strengthen science; third, we need to remodel, diversify, and secure research careers; and finally, we need to unite and coordinate efforts for change.

## Introduction

To succeed in science, researchers need to maximize the number of grants they receive, the number of articles they publish, and the bibliometric impact they obtain on their scientific output. These achievements are generally measured with narrow and decontextualized metrics that fail to capture creativity, innovation, openness, and quality [1]. Demands for high-competition, high-output, and high-impact research can also lead to poor quality research and research waste, inspire breaches of research integrity, and tax the wellbeing of researchers.

## Main text

**Fig. 1:**
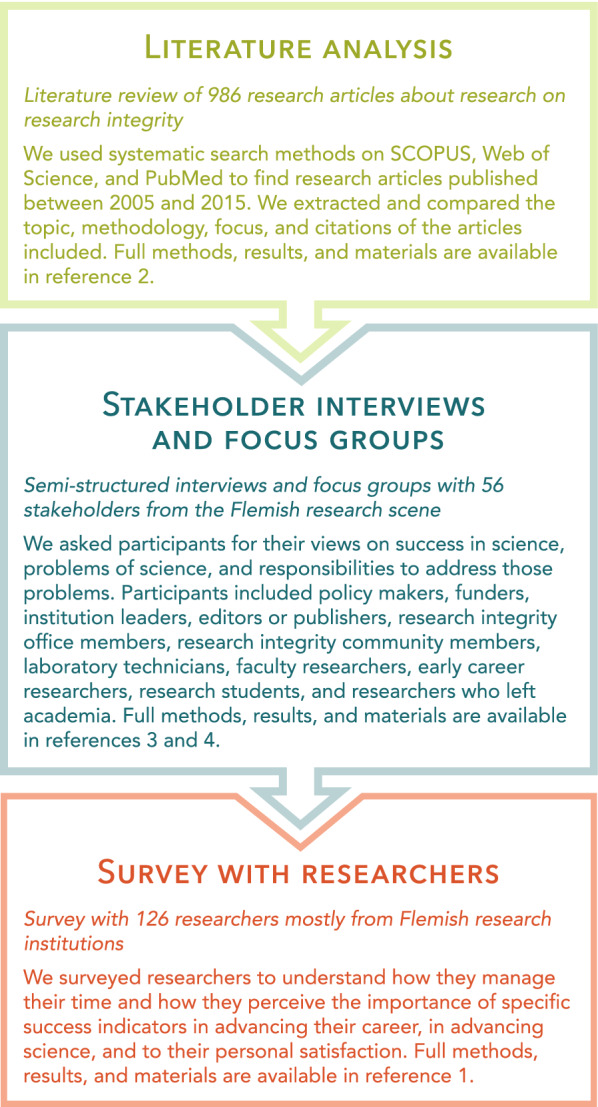
The three core methodologies used to gather our findings

**Fig. 2:**
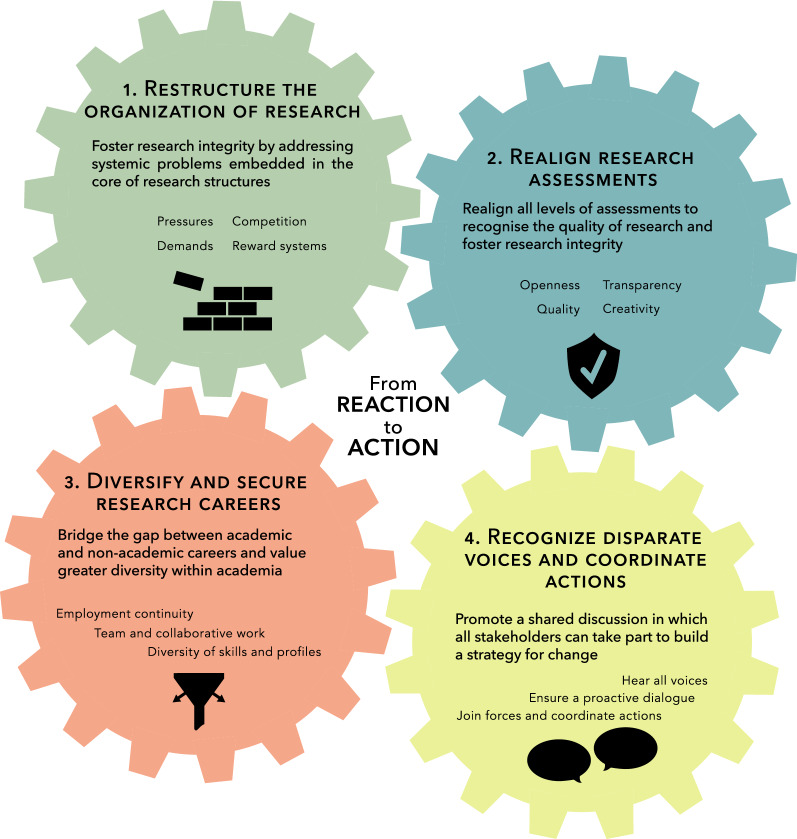
Four recommendations to help overcome the current problems of science

In the past 5 years, we captured the perspectives of a broad array of those with a stake in the production of scientific knowledge to better understand the impact that our current approach to success may have on the integrity and the quality of research. Our findings–based on a thorough literature analysis [2], stakeholder interviews and focus groups [3, 4], and a survey of researchers [1] (see Fig. 1)–provide new insights that translate to four recommendations for overcoming the current problems that plague research: restructuring the organization of research, realigning research assessments, remodeling research careers, and recognizing and coordinating efforts to move from reaction to action (Fig. 2). The full methods, results, and materials used in the different steps of our research are published in separate papers [1–4], and further material is available on the Open Science Framework [5].

### Restructure the organization of research

Our project began with an analysis of the literature on research integrity, where we found a disconnect between evidence and practice. While research integrity is most often shown to be disturbed by issues within research systems such as competition, pressure, and incentives, approaches to foster integrity generally focus on researchers’ awareness and compliance and not on the systemic problems of academia [2].

Without discrediting the value of integrity training, codes of conduct, whistleblowing protection, and oversight in building a solid culture of integrity among researchers, our findings show that the promotion of research integrity requires interventions that address systemic imperfections of the research enterprise, including, most importantly, the incentives and reward structures of research.

### Realign research assessments

Echoing statements such as the San Francisco Declaration on Research Assessment (DORA; 6), the Leiden Manifesto for research metrics [7], The Metric Tide [8], and the Hong Kong Principles for Assessing Researchers [9], our findings show that research assessments must change in a way that values and fosters the integrity and the quality of research. We found, for example, that researchers believe that the indicators now being used to assess research careers do not align with the indicators that are important for advancing science [1]. This finding agrees with a broad body of research that demonstrates the inadequacy of current indicators for capturing social impact [10, 11], innovation [12], replicability [13], and quality [14].

Furthermore, an overemphasis on outputs, quantity, and ground-breaking results discourages high quality research and overlooks the importance of negative results and the need for replication in research [4]. The increasing prominence of project-based research funding further deepens the problem by exacerbating pressures on individuals and by giving a short-term mindset to research processes. Our findings provide empirical support to the strong momentum for change visible in ongoing efforts to encourage more responsible use of metrics, broader consideration of diverse research activities, and greater recognition of processes such as quality, openness, and transparency (many of these ongoing initiatives are described in 15, 16). But realigning research assessments also requires interventions that go beyond changes in research institutions. Indeed, performance-based research funding and university rankings at national and international levels have a powerful influence on the perspectives of success and excellence [17, 18] and realigning these high-level assessments with integrity and high quality research is equally important in improving science.

### Diversify and secure research careers

It is also important to consider the person behind the research. At the moment, only ten to twenty percent of PhD students will be able to secure a permanent position in academia although most aspire to an academic career [19–22]. This is not a new problem. The issue has been raised for more than twenty years with very little change [23, 24]. One of the strengths of our project was the inclusion of past researchers who have found careers outside academia. In hearing their stories, we understood that leaving academia can generate a vivid wound and leave a strong feeling of failure [3]. The scarce opportunities for employment also increase pressure and competition between early career researchers thus isolating them, jeopardizing their mental health [25, 26], and requiring them to outpace their colleagues to survive in their academic career. To move ahead of colleagues, researchers need to focus on outputs and ignore processes that are not rewarded, even if many of these processes are essential in advancing science [4]. Highly selective research careers also block diversity, not only in terms of gender and ethnicity, but also in terms of skills and career profiles of those who succeed. As a result, academic research environments are shaped by a uniform research culture that is highly resistant to change. There is an urgent need to address research careers and employment insecurity. Research institutions and doctoral schools need to provide early career researchers with better opportunities to develop transferable skills and connect with non-academic sectors. But academic careers themselves would also benefit from greater differentiation, including diverse roles within academia where unique skills and profiles are acknowledged, incentivized, and rewarded and where collaborative teams and diverse interpretations of success are considered [27].

### Recognize disparate voices and coordinate actions

Our project involved a wide array of stakeholders including policy makers, research funders, research institution leaders, editors and publishers, research integrity office members, early-, mid-, and late-career researchers, research students, laboratory technicians, and researchers who left academia. In hearing the voices of so many different stakeholders, we realized that perspectives of success, integrity, and misconduct differ between individuals and that the problems and actions needed are interpreted differently by different stakeholders. We also found that the responsibility for actions is often passed from one actor to the next, creating a stagnant system characterized by blame, hopelessness, and inaction [3]. Despite this discouraging picture, the past few years have seen an emergence of working groups and networks of researchers eager to change and move from discussion to action. With over 20,000 signatories–2500 of which are organizations–the San Francisco Declaration on Research Assessments is a perfect example of the emergent mobilization, a movement that has captured the attention of important funders such as the Wellcome Trust in the UK, the Canadian federal Tri-Agency, and the Australian National Health and Medical Research Council, among many others. The dialogue is also increasingly diverse, merging the voices of different stakeholders who are willing to join forces to make research better [15]. But the voices of former-researchers and early career scientists whose perspectives may be very different than that of those who survived and succeeded in the current system are often missed. For broad, systemic changes to be operationalized, we need to understand the dynamics and the relationships at play in the current problems as experienced by *all* actors involved. We need to dig deeper in the spaces and responsibilities that link different actors and that build the foundations of our shared concepts of excellence and integrity. Broad expert groups such as the European Commission Policy Platforms or expert groups created by Scientific Societies and Academies, for example, provide a venue where the opinions of different actors meet and influence those who make science policy. Ensuring that these platforms include the full diversity of voices is the next logical step to ensuring a proactive dialogue.

### From reaction to action

These four recommendations suggest that the very foundations of research systems need to be addressed. Although daunting, we are confident that change is possible. Over the course of the 5 years of this project, much has happened in the field of research integrity and especially in the area of research assessment. As we were conducting our research, new developments, assessment initiatives, position documents, and influential opinions on the topic were emerging nearly every week, giving us great hope for the future. Nevertheless, our research suggests that topical initiatives will only realize their full potential and change research culture if they generate broad and coordinated approaches for change. Recent actions in this direction are promising. Hints at global change can be found in the recent ‘Agreement on Reforming Research Assessment’ supported by the European Commission, Science Europe, and the European University Association [28], in the Global Research Council ‘Responsible Research Assessment – Call to Action’ [29], and in statements from wide-reaching multi-stakeholder programs such as the ‘G7 2021 Research Compact’ [30] and the ‘UNESCO Recommendation on Open Science’ [31]. Now is the time for a shift from *discussing* what needs to change to *enacting* change.

## Limitations

The results from the focus groups, interviews, and survey reported in this short Research Note principally came from stakeholders involved with Flemish (Belgium) biomedical research. For this reason, some of the results may be specifically relevant to Flemish research or to biomedical sciences, and may not apply to different settings. However, the high compatibility of our findings with current research and policy efforts (see for example 8, 32, 33–38) suggests that different settings and disciplines share a similar perspective of the problems and changes needed than the participants of our research. Additional and more detailed limitations for the different empirical steps reported in this Research Note are available in the respective papers in which the full findings are reported [1–4].

## Data Availability

The data that support the findings from the survey study are available in the associated paper (see 1). The data that supports the results from the interviews and focus groups study are not publicly available due to the risk of identification of participants, but extensive quotes are available in the papers describing the findings [3, 4]. The survey and interview or focus group guides used to gather the data are available in their associated papers [1, 3, 4], and additional material such as consent forms and participant information sheet are available in the Open Science Framework (5).

## Abbreviation

DORA: San Francisco declaration on research assessment
